# Recent developments in Alzheimer's disease therapeutics

**DOI:** 10.1186/1741-7015-7-7

**Published:** 2009-02-19

**Authors:** Michael S Rafii, Paul S Aisen

**Affiliations:** 1 Department of Neurosciences, University of California San Diego, Gilman Drive M/C 0949, La Jolla, CA 92093, USA

## Abstract

Alzheimer's disease is a devastating neurological disorder that affects more than 37 million people worldwide. The economic burden of Alzheimer's disease is massive; in the United States alone, the estimated direct and indirect annual cost of patient care is at least $100 billion. Current FDA-approved drugs for Alzheimer's disease do not prevent or reverse the disease, and provide only modest symptomatic benefits. Driven by the clear unmet medical need and a growing understanding of the molecular pathophysiology of Alzheimer's disease, the number of agents in development has increased dramatically in recent years. Truly *disease-modifying' therapies that target the underlying mechanisms of Alzheimer's disease have now reached late stages of human clinical trials. Primary targets include beta-amyloid, whose presence and accumulation in the brain is thought to contribute to the development of Alzheimer's disease, and tau protein which, when hyperphosphorylated, results in the self-assembly of tangles of paired helical filaments also believed to be involved in the pathogenesis of Alzheimer's disease. In this review, we briefly discuss the current status of Alzheimer's disease therapies under study, as well the scientific context in which they have been developed.

## Review

Synaptotoxic β-amyloid (Aβ) peptide and the plaques composed of aggregated Aβ, as well as the neurofibrillary tangles composed of hyperphosphorylated tau protein, are believed to be central to the pathogenesis of Alzheimer's disease (AD). Although both the amyloid and tangle pathways present multiple opportunities to create disease-modifying therapies for AD, most of the biotech and pharmaceutical industry efforts have focused on the *amyloid hypothesis'; this focus is supported by strong genetic evidence implicating the primacy of the amyloid pathway [1].

Therapeutic strategies aimed at preventing Aβ formation, blocking its aggregation into plaques, lowering its soluble levels in the brain, and disassembling existing amyloid plaques are among the main strategies employed to slow the progression of AD. Recently, a few therapeutic programs have aimed at reducing tau phosphorylation and/or aggregation. Beyond plaque- and tangle-related targets, other aspects of AD pathophysiology, including mitochondrial dysfunction, failure of molecular transport mechanisms, oxidative damage, inflammation, and cell-cycle dysregulation, may also provide therapeutic opportunities. In this review, we will discuss drugs under study that are thought to slow the progression of AD by each of the above-mentioned mechanisms. Key development programs are listed in Table 1.

**Table 1:** Selected Alzheimer's disease drug development programs

**Drug**	**Mechanism of Action**	**Stage of Development**
Tramiprosate	Direct Aβ binding to prevent Aβ aggregation	Completed Phase III/discontinued

ACC-001	Active Aβ vaccination	Phase II (safety, proof of concept)

Bapineuzumab	Anti-Aβ monoclonal antibodies	Phase III (efficacy in AD)

IgIV	Anti-Aβ polyclonal antibodies	Phase III (efficacy in AD)

PF-04494700	RAGE Inhibitor	Phase II (safety, proof of concept)

Tarenflurbil	γ-secretase modulator	Completed Phase III/discontinued

Semagacestat	γ-secretase inhibitor	Phase III (efficacy in AD)

Rember	Tau aggregation inhibitor	Entering Phase III (efficacy in AD)

NAP (AL-108)	Microtubule stabilizer	Phase II (safety, cognitive enhancement)

Dimebon	Mitochondrial stabilizer	Phase III (efficacy in AD)

### Decreasing Aβ formation through direct binding or altered trafficking

Direct interaction with Aβ may reduce aggregation and accumulation, thereby limiting amyloid-mediated synaptic dysfunction and neurotoxicity.

#### Tramiprosate

The first anti-amyloid drug to reach a pivotal clinical trial was tramiprosate. Tramiprosate is a glycosaminoglycan mimetic that binds to monomeric Aβ, thereby reducing aggregation and neurotoxicity while promoting clearance from brain [2]. A Phase II trial demonstrated that the drug reduces Aβ42 in the cerebrospinal fluid of patients with AD [3]. The North American Phase III study included 1052 patients with mild to moderate AD, randomly assigned to receive placebo or 100 mg or 150 mg twice daily of tramiprosate. Though treatment was well tolerated, the study failed to demonstrate a beneficial effect on the primary outcomes, change in cognition and clinical stage. Unexplained variance in the planned statistical model may have contributed to these disappointing results. There are currently no additional trials planned utilizing this mechanism of action.

#### Abeta42 vaccines, monoclonal Aβ antibodies, polyclonal antibodies

Immunotherapy targeting the amyloid peptide is a leading approach to disease-modifying treatment [4]. Mechanistically, molecules that bind Aβ peptide in the blood could *draw' the peptide from the brain through the blood-brain barrier, possibly by a receptor-mediated process. Heparin, gelsolin, and other molecules are thought to *sink' or trap Aβ peptide in the blood and, at least in animal models, reduce Aβ accumulation in the brain [5]. Alternatively, such antibodies, which generally penetrate into the brain to a small but definite extent, may promote microglial phagocytosis and clearance of amyloid.

A Phase II trial of the first-generation amyloid vaccine AN-1792 (Elan/Wyeth), using aggregated amyloid peptide as the immunogen, suggested a positive efficacy trend: patients with AD who developed an antibody response improved over a 12-month period in some neuropsychological tests [6]. However, owing to the development of aseptic meningoencephalitis in 6% of the patients the AN-1792 program was discontinued. A second-generation vaccine, ACC-001 (Elan/Wyeth), which was engineered to have an improved safety profile (with a short Aβ sequence as the immunogen, presumably preventing the induction of a toxic cellular immune response), is now in a Phase II clinical trial. Similar active vaccination programs from Novartis and Merck are also in clinical testing.

Compared with active immunization, passive immunization would incorporate regular intravenous administration of anti-Aβ antibodies which, although cumbersome, could offer more control over safety and efficacy. A number of monoclonal antibodies against various domains of Aβ are currently in development to capitalize on this notion. The furthest along in clinical testing is bapineuzumab (Elan/Wyeth), now being investigated in a series of Phase III trials; a Phase II study of this antibody yielded some encouraging results, particularly in the subgroup of patients not carrying the apolipoprotein ε4 allele. A small Phase II trial of pooled human immunoglobulin (IgIV) which contains naturally occurring anti-amyloid antibodies showed evidence of cognitive and clinical benefits, and a Phase III trial has just been launched.

#### RAGE Inhibitor

Amyloid is also known to bind to receptors for advanced glycated endproducts (RAGE) on the surface of cells and at the blood-brain barrier; this binding may contribute to inflammation and neuronal death [7]. In laboratory studies, blocking amyloid-RAGE binding can reduce amyloid accumulation and neurotoxicity. PF-04494700 (formerly TTP488) is an orally bioavailable small molecule antagonist of RAGE. It is now being investigated in a Phase II clinical study to determine its potential in AD therapy.

### Decreasing Aβ production – gamma-secretase inhibitors

Another approach to lowering the level of Aβ in the brain is to decrease its production. Gamma-secretase is a transmembrane enzyme complex that cleaves the amyloid precursor protein at one end of the Aβ sequence; its activity is required for Aβ generation in brain.

#### Tarenflurbil

Tarenflurbil, the enantiomer of the non-steroidal anti-inflammatory drug flurbiprofen, modulates the activity of gamma-secretase, thereby reducing Aβ [8]. In a Phase II trial in patients with mild-to-moderate AD, tarenflurbil was safe and well tolerated, but the primary analyses did not show a beneficial effect on cognition or function; post-hoc analyses suggested possible benefit of treatment of mildly affected patients with the highest dose tested [9]. A large 18-month Phase III trial showed no benefit of treatment, and the development program has been discontinued. A plausible explanation for the failure of tarenflurbil is that oral administration produced insufficient brain concentrations to reduce Aβ to a meaningful extent.

#### Other gamma-secretase inhibitors

A large Phase III study examining treatment with semagacestat (LY450139) [10], a gamma-secretase inhibitor, is currently underway. Earlier studies of this drug demonstrated significant reduction of amyloid peptide generation in blood and cerebrospinal fluid of patients with AD treated with tolerable doses; this is notable in view of concerns that gamma-secretase inhibition may cause serious toxicity mediated by inhibition of Notch cleavage [11].

A number of other candidate anti-amyloid compounds are entering clinical trials. These include inhibitors of beta-secretase (the second endopeptidase involved in Aβ cleavage from its precursor protein), considered by some to be the most promising target of all because it may be an essential component of the amyloid cascade yet its inhibition may be free of serious toxicity [12]. Additional anti-aggregation agents such as scyllo-inositol [13] and compounds that interfere with the interaction of amyloid peptides with metals [14] have also shown some promise.

### Targeting tau

Consensus is growing that a truly effective, disease-modifying therapy will have to reduce both amyloid and tau-related pathology. Tau is a microtubule-associated protein, abundant in neurons, which promotes and stabilizes tubulin assembly into microtubules. Hyperphosphorylation of tau interferes with its function and can result in self-assembly into paired helical filaments that form intraneuronal tangles. In the neurobiology underlying the development of AD, there is a progression of hyperphosphorylated tau-bearing tangles appearing initially in the entorhinal cortex and hippocampus, then progressing to neocortex of the temporal, frontal, and parietal cortices. An optimal disease-modifying strategy should interfere with this cascade either by interfering with an upstream causal event (thought by many to be generation of Aβ) or targeting tangle formation directly.

#### Methylene blue

Methylene blue, a widely used histology dye, has been shown to interfere with tau aggregation [15]. This compound is now being investigated (under the trade name Rember) as a potential treatment for AD. A Phase II study has been completed; some analyses suggested a drug benefit in subsets of participants [16]. Pivotal Phase III trials are planned, with the aim of providing definitive evidence of the efficacy and safety of this approach.

#### NAP

A small peptide called NAP (AL-108), derived from a natural neurotrophic protein, can be delivered to the central nervous system via intranasal administration [17]. Animal studies indicate that intranasal NAP markedly reduces tau phosphorylation [18], and preliminary human studies have been encouraging.

### Neuroprotection

The term neuroprotection refers to mechanisms that protect neurons from degeneration, for example following ischemic injury or as a result of chronic neurodegenerative diseases. AD and other neurodegenerative disorders are associated with oxidative and inflammatory stress and mitochondrial dysfunction. While trials of antioxidants and anti-inflammatory treatments have provided modest or no beneficial effects, efforts to develop effective neuroprotectants continue.

#### Dimebon

Dimebon is a 25-year-old antihistamine that was studied in Russia as a treatment for AD on the basis of *in vitro *evidence of cholinesterase inhibition and NMDA receptor antagonism; in fact, the efficacy of Dimebon in AD appears to be unrelated to these activities, but relates rather to a unique mechanism of action involving stabilization of mitochondria. In a 6-month trial of Dimebon (20 mg three times a day) in 183 patients with mild to moderate AD conducted in Russia, the drug showed significant improvement in all cognitive, behavioral and global outcome measures [19]. Further, the benefits of drug treatment were greater after a blinded extension period of 6 months, suggesting the possibility of a disease-modifying effect. The sponsor, Medivation, is now conducting a confirmatory Phase III trial.

## Conclusion

Currently, the anti-amyloid strategies are proceeding with the greatest number of candidate drugs. Numerous candidate disease-modifying therapies that target the underlying pathogenic mechanisms of AD are currently in clinical trials. While it is not possible to predict the success of any individual program, one or more are likely to prove effective. Indeed, it seems reasonable to predict that in the not-too-distant future, a synergistic combination of agents will have the capacity to alter the neurodegenerative cascade and reduce the global impact of this devastating disease.

## Competing interests

MSR has nothing to disclose. PSA is the recipient of grants from Pfizer and Baxter, and is a consultant to Elan, Wyeth, Eisai, Novartis, Neurochem, Schering-Plough, Bristol Myers Squibb, Lilly, Medivation, Neurophage, Merck and Roche.

## Authors' contributions

MSR and PSA each participated in writing and editing of this minireview. Both authors have read and approved the final manuscript.

## Pre-publication history

The pre-publication history for this paper can be accessed here:



## References

[B1] Hardy J, Selkoe DJ (2002). The amyloid hypothesis of Alzheimer's disease: progress and problems on the road to therapeutics. Science.

[B2] Gervais F (2004). GAG mimetics: potential to modify underlying disease process in AD. Neurobiol Aging.

[B3] Aisen PS, Saumier D, Briand R, Laurin J, Gervais F, Tremblay P, Garceau D (2006). A Phase II study targeting amyloid-beta with 3APS in mild-to-moderate Alzheimer disease. Neurology.

[B4] Relkin NR (2008). Current state of immunotherapy for Alzheimer's disease. CNS Spectr.

[B5] DeMattos RB, Bales KR, Cummins DJ, Paul SM, Holtzman DM (2002). Brain to plasma amyloid-beta efflux: a measure of brain amyloid burden in a mouse model of Alzheimer's disease. Science.

[B6] Gilman S, Koller M, Black RS, Jenkins L, Griffith SG, Fox NC, Eisner L, Kirby L, Rovira MB, Forette F, Orgogozo JM (2005). Clinical effects of Abeta immunization (AN1792) in patients with AD in an interrupted trial. Neurology.

[B7] Chen X, Walker DG, Schmidt AM, Arancio O, Lue LF, Yan SD (2007). RAGE: a potential target for Abeta-mediated cellular perturbation in Alzheimer's disease. Curr Mol Med.

[B8] Eriksen JL, Sagi SA, Smith TE, Weggen S, Das P, McLendon DC, Ozols VV, Jessing KW, Zavitz KH, Koo EH, Golde TE (2003). NSAIDs and enantiomers of flurbiprofen target gamma-secretase and lower Abeta 42 in vivo. J Clin Invest.

[B9] Wilcock GK, Black SE, Hendrix SB, Zavitz KH, Swabb EA, Laughlin MA (2008). Efficacy and safety of tarenflurbil in mild to moderate Alzheimer's disease: a randomised phase II trial. Lancet Neurol.

[B10] Fleisher AS, Raman R, Siemers ER, Becerra L, Clark CM, Dean RA, Farlow MR, Galvin JE, Peskind ER, Quinn JF, Sherzai A, Sowell BB, Aisen PS, Thal LJl (2008). Phase 2 safety trial targeting amyloid beta production with a gamma-secretase inhibitor in Alzheimer disease. Arch Neurol.

[B11] Wolfe MS (2008). Inhibition and modulation of gamma-secretase for Alzheimer's disease. Neurotherapeutics.

[B12] Ghosh AK, Gemma S, Tang J (2008). beta-Secretase as a therapeutic target for Alzheimer's disease. Neurotherapeutics.

[B13] McLaurin J, Golomb R, Jurewicz A, Antel JP, Fraser PE (2000). Inositol stereoisomers stabilize an oligomeric aggregate of Alzheimer amyloid beta peptide and inhibit abeta-induced toxicity. J Biol Chem.

[B14] Lannfelt L, Blennow K, Zetterberg H, Batsman S, Ames D, Harrison J, Masters CL, Targum S, Bush AI, Murdoch R, Wilson J, Ritchie CW (2008). Safety, efficacy, and biomarker findings of PBT2 in targeting Abeta as a modifying therapy for Alzheimer's disease: a phase IIa, double-blind, randomised, placebo-controlled trial. Lancet Neurol.

[B15] Wischik CM, Edwards PC, Lai RY, Roth M, Harrington CR (1996). Selective inhibition of Alzheimer disease-like tau aggregation by phenothiazines. Proc Natl Acad Sci USA.

[B16] Gura T (2008). Hope in Alzheimer's fight emerges from unexpected places. Nat Med.

[B17] Gozes I, Morimoto BH, Tiong J, Fox A, Sutherland K, Dangoor D, Holser-Cochav M, Vered K, Newton P, Aisen PS, Matsuoka Y, van Dyck CH, Thal L (2005). NAP: research and development of a peptide derived from activity-dependent neuroprotective protein (ADNP). CNS Drug Rev.

[B18] Matsuoka Y, Jouroukhin Y, Gray AJ, Ma L, Hirata-Fukae C, Li HF, Feng L, Lecanu L, Walker BR, Planel E, Arancio O, Gozes I, Aisen PS (2008). A neuronal microtubule-interacting agent, NAPVSIPQ, reduces tau pathology and enhances cognitive function in a mouse model of Alzheimer's disease. J Pharmacol Exp Ther.

[B19] Doody RS, Gavrilova SI, Sano M, Thomas RG, Aisen PS, Bachurin SO, Seely L, Hung D (2008). Effect of dimebon on cognition, activities of daily living, behaviour, and global function in patients with mild-to-moderate Alzheimer's disease: a randomised, double-blind, placebo-controlled study. Lancet.

